# Transfluthrin eave-positioned targeted insecticide (EPTI) reduces human landing rate (HLR) of pyrethroid resistant and susceptible malaria vectors in a semi-field simulated peridomestic space

**DOI:** 10.1186/s12936-021-03880-2

**Published:** 2021-08-30

**Authors:** Mgeni M. Tambwe, Sarah Moore, Lorenz Hofer, Ummi A. Kibondo, Adam Saddler

**Affiliations:** 1 Vector Control Product Testing Unit (VCPTU), Environmental Health and Ecological Sciences, Ifakara Health Institute, P.O. Box 74, Bagamoyo, Tanzania; 2 Health Interventions Unit, Department of Epidemiology & Public Health, Swiss Tropical & Public Health Institute, Socinstrasse 57, 4051 Basel, Switzerland; 3 University of Basel, Petersplatz 1, 4001 Basel, Switzerland; 4 Telethon Kids Institute, Perth, Australia

**Keywords:** Volatile pyrethroid, Transfluthrin, Pyrethroid resistance, Eave-positioned targeted insecticide, EPTI, *Anopheles gambiae*, *Anopheles arabiensis*, Semi-field system

## Abstract

**Background:**

Volatile pyrethroids (VPs) are proven to reduce human–vector contact for mosquito vectors. With increasing resistance to pyrethroids in mosquitoes, the efficacy of VPs, such as transfluthrin, may be compromised. Therefore, experiments were conducted to determine if the efficacy of transfluthrin eave-positioned targeted insecticide (EPTI) depends on the resistance status of malaria vectors.

**Methods:**

Ribbons treated with 5.25 g transfluthrin or untreated controls were used around the eaves of an experimental hut as EPTI inside a semi-field system. Mosquito strains with different levels of pyrethroid resistance were released simultaneously, recaptured by means of human landing catches (HLCs) and monitored for 24-h mortality. Technical-grade (TG) transfluthrin was used, followed by emulsifiable concentrate (EC) transfluthrin and additional mosquito strains. Generalized linear mixed models with binomial distribution were used to determine the impact of transfluthrin and mosquito strain on mosquito landing rates and 24-h mortality.

**Results:**

EPTI treated with 5.25 g of either TG or EC transfluthrin significantly reduced HLR of all susceptible and resistant *Anopheles* mosquitoes (Odds Ratio (OR) ranging from 0.14 (95% Confidence Interval (CI) [0.11–0.17], P < 0.001) to 0.57, (CI [0.42–0.78] *P* < 0.001). Both TG and EC EPTI had less impact on landing for the resistant *Anopheles arabiensis* (Mbita strain) compared to the susceptible *Anopheles gambiae* (Ifakara strain) (OR 1.50 [95% CI 1.18–1.91] *P* < 0.001) and (OR 1.67 [95% CI 1.29–2.17] *P* < 0.001), respectively. The EC EPTI also had less impact on the resistant *An. arabiensis* (Kingani strain) (OR 2.29 [95% CI 1.78–2.94] *P* < 0.001) compared to the control however the TG EPTI was equally effective against the resistant Kingani strain and susceptible Ifakara strain (OR 1.03 [95% CI 0.82–1.32] *P* = 0.75). Finally the EC EPTI was equally effective against the susceptible *An. gambiae* (Kisumu strain) and the resistant *An. gambiae* (Kisumu-kdr strain) (OR 0.98 [95% CI 0.74–1.30] *P* = 0.90).

**Conclusions:**

Transfluthrin-treated EPTI could be useful in areas with pyrethroid-resistant mosquitoes, but it remains unclear whether stronger resistance to pyrethroids will undermine the efficacy of transfluthrin. At this dosage, transfluthrin EPTI cannot be used to kill exposed mosquitoes.

**Supplementary Information:**

The online version contains supplementary material available at 10.1186/s12936-021-03880-2.

## Background

Indoor residual spraying (IRS) and long-lasting insecticidal nets (LLINs) are currently the core mosquito vector control tools employed in national malaria control programmes worldwide [1]. Since 2000, global malaria incidence has decreased by 37% and mortality by 60% [2], to which these tools have contributed approximately 70% of the reduction [1]. However, there are concerns that progress has stagnated  and malaria increased in several countries between 2015 and 2019 [3]. Increased transmission in some areas where elimination was considered to be feasible has also been observed [4, 5]. This increase is likely caused by insufficient coverage and use of core interventions, with fewer than half of households in sub-Saharan Africa owning enough nets for all occupants [3]. Progress may also be impeded by limitations of the core interventions and their effectiveness in certain settings. For example, the current tools do not provide protection in outdoors setting where humans and vectors frequently come into contact before bed time [6]. Furthermore, the development of physiological resistance [7] in mosquito vectors may undermine the continued efficacy of IRS and LLINs [8].

Development of alternative control strategies that cover the existing gaps and that compliment core control tools remains necessary [9]. Proposed measures include spatial repellents (SR) [10, 11], genetically engineered mosquitoes [12], attractive targeted (toxic) sugar bait (ATSB) [13] and endectocides, such as ivermectin [14]. The focus of this study is SR from the pyrethroid class often referred to as volatile pyrethroids (VPs). Volatile pyrethroids vaporize at room temperature and are dispersed into the surrounding area with the aim of creating a bite-free space [15], and they can be used indoors and outdoors. Previous studies have demonstrated that VPs, such as transfluthrin and metofluthrin, are effective at reducing the human landing rate (HLR) for a range of mosquitoes [16]. Passive emanators treated with transfluthrin or metofluthrin consistently demonstrated personal protective efficacy exceeding 50% in studies conducted in Cambodia [17], Tanzania [18], Belize [19] and Indonesia [20]. Transfluthrin applied to hessian strips as eave-positioned targeted insecticide (EPTI) has provided over 68% reduction in human vector contact in semi-field studies [10, 21] and over 80% in field studies in Tanzania [10, 11]. Volatile pyrethroids exhibit a dose response, with lower concentrations eliciting behavioural effects that include deterrence, excito-repellency and blood-feeding inhibition [22] and with higher concentrations or longer exposure times increasing knockdown and mortality [23].

Pyrethroid have been the main class of insecticide used in LLINs and IRS [24]. Resistance to these insecticides is now widespread [25], which poses a threat not only to the efficacy of LLINs and IRS but potentially also to VPs. Furthermore, effective, long-lasting volatile insecticides of chemical classes other than pyrethroids are not yet available for public health use [26]. It is necessary to know whether the efficacy of VPs may be compromised by pyrethroid resistance and, therefore, if VPs can be used in areas with existing pyrethroid-resistant mosquito populations. VPs are from the same chemical class, which would normally indicate cross-resistance; however, structural differences between transfluthrin and non-volatile pyrethroid indicate that cross-resistance may not occur [27]. Therefore, the objectives of this study were to determine (1) the efficacy of transfluthrin applied as EPTI to reduce HLR of multiple strains of Afrotropical malaria vectors with varying levels of pyrethroid resistance and (2) delayed mortality induced by EPTI exposure.

## Methods

### Study site

The experiment was conducted in a semi-field system (SFS) located in Bagamoyo, Tanzania, from March 2018 to October 2018 and from August 2019 to September 2019. The SFS measures 21 × 29 × 4.5 m and is divided into three compartments. Two heavy-duty polyethylene walls separate these compartments, preventing air movement between the chambers and reducing the chance of cross-contamination when working with VPs or other aerosols. The SFS allows for controlled experiments with disease-free mosquitoes to be conducted under field like climatic conditions [28]. In each compartment, an experimental hut [29] was constructed, and tests were conducted outside the huts to simulate a peridomestic space.

### Study mosquitoes

Five laboratory-reared mosquito strains were used in these experiments: (1) pyrethroid-susceptible *Anopheles gambiae *sensu stricto (s.s.) (Kisumu strain) and (2) pyrethroid-resistant *An. gambiae* s.s. (Kisumu-*kdr* strain) with L1014S *kdr*, i.e., *kdr*-east resistance mechanism [30], both originating from Kisumu, Kenya; (3) pyrethroid-susceptible *An. gambiae* s.s. (Ifakara strain) originating from Ifakara, Tanzania, and in colony at IHI since 1996; (4) pyrethroid-resistant *Anopheles arabiensis* (Mbita strain) from the International Centre of Insect Physiology and Ecology (ICIPE), Kisumu, Kenya, expressing a moderate level of phenotypical resistance against permethrin and deltamethrin (the mechanism is likely metabolic but not confirmed); and (5) *An. arabiensis* (Kingani strain) originating from Ifakara and in colony at Bagamoyo since 2015, expressing a high level of phenotypical resistance against permethrin and deltamethrin [31]. The two *An. arabiensis* strains have been tested and found to be free of *kdr* mutations (L1014F *kdr-west* and L1014S *kdr-east*) (unpublished data) commonly associated with pyrethroid resistance. It is likely that the metabolic resistance mechanism was responsible for their survival in the presence pyrethroid insecticides.

Before the start of semi-field experiments, susceptibility tests were conducted for each mosquito strain using tube test bioassays performed following World Health Organization (WHO) guidelines [32]. Non-blood-fed 3- to 5-day-old mosquitoes were exposed to insecticide-impregnated papers at the standard WHO discriminating dose for the pyrethroids permethrin (0.75%) and deltamethrin (0.05%). These insecticides were selected because they belong to the same chemical class as transfluthrin and are commonly used on LLINs.

All mosquito strains are maintained at the Bagamoyo branch of the Ifakara Health Institute (IHI) according to MR4 guidelines [33]. Larvae are fed on fish food (TetraMin® tropical flakes) and adult mosquitoes on 10% sucrose ad libitum. Bovine blood meals are provided to adult females for egg production using membrane-feeding assay. The insectary is maintained at 27 ± 5 °C and 70–100% relative humidity with approximately 12:12 light:dark (ambient lighting).

The experiments used 3- to 8-day-old female mosquitoes that had never blood-fed. The mosquitoes were sugar starved for 6 h prior to the experiment. Because more than one mosquito strain with the same morphology was released simultaneously, red and yellow fluorescent pigments (Swada, Cheshire, UK) were used to differentiate between strains. Mosquitoes were marked in a cup by dusting the mesh lid of the cup with a brush containing the colour pigment; thereby creating a cloud of pigment that was transferred to the mosquitoes in small amounts. Preliminary experiments indicated that the fluorescent pigments did not influence mosquito responses, feeding behaviours or survival. Also the same fluorescent has been used in the marking and recapture experiment without altering the behaviour of the coloured mosquitoes [34].

### Preparation of transfluthrin eave-positioned targeted insecticide (EPTI)

Hessian material has proved very useful for the delivery of transfluthrin because it has a much slower release rate than other textiles and thus increases the longevity of the VP devices [21, 35, 36]. Hessian sacks were purchased locally, washed using well water and powder detergent (OMO®, Unilever, Nairobi, Kenya), dried under direct sunlight and then cut into 21 m × 10 cm strips. The hessian was treated with either TG or EC transfluthrin formulations (Bayothrin EC, Bayer AG, Monheim am Rhein, Germany). The experiments were initially conducted using TG transfluthrin emulsified with 100 ml of Tween®20 (Sigma-Aldrich, CAS #9005-64-5). Bayer developed and introduced EC transfluthrin that was used for further experiments. In all experiments, with either formulation, 5.25 g of transfluthrin was impregnated into hessian equivalent to 2.5 g/m^2^. Drying took place out of direct sunlight to protect the transfluthrin from photolysis by exposure to ultraviolet light [27, 37]. For the control arms, the strips were prepared in the same manner as the treated strips but with only water. During the day, the treated hessian was kept out of direct sunlight at the ambient outdoor temperature (24–27.6 °C) on a metal frame.

### Experimental procedure

**Fig. 1:**
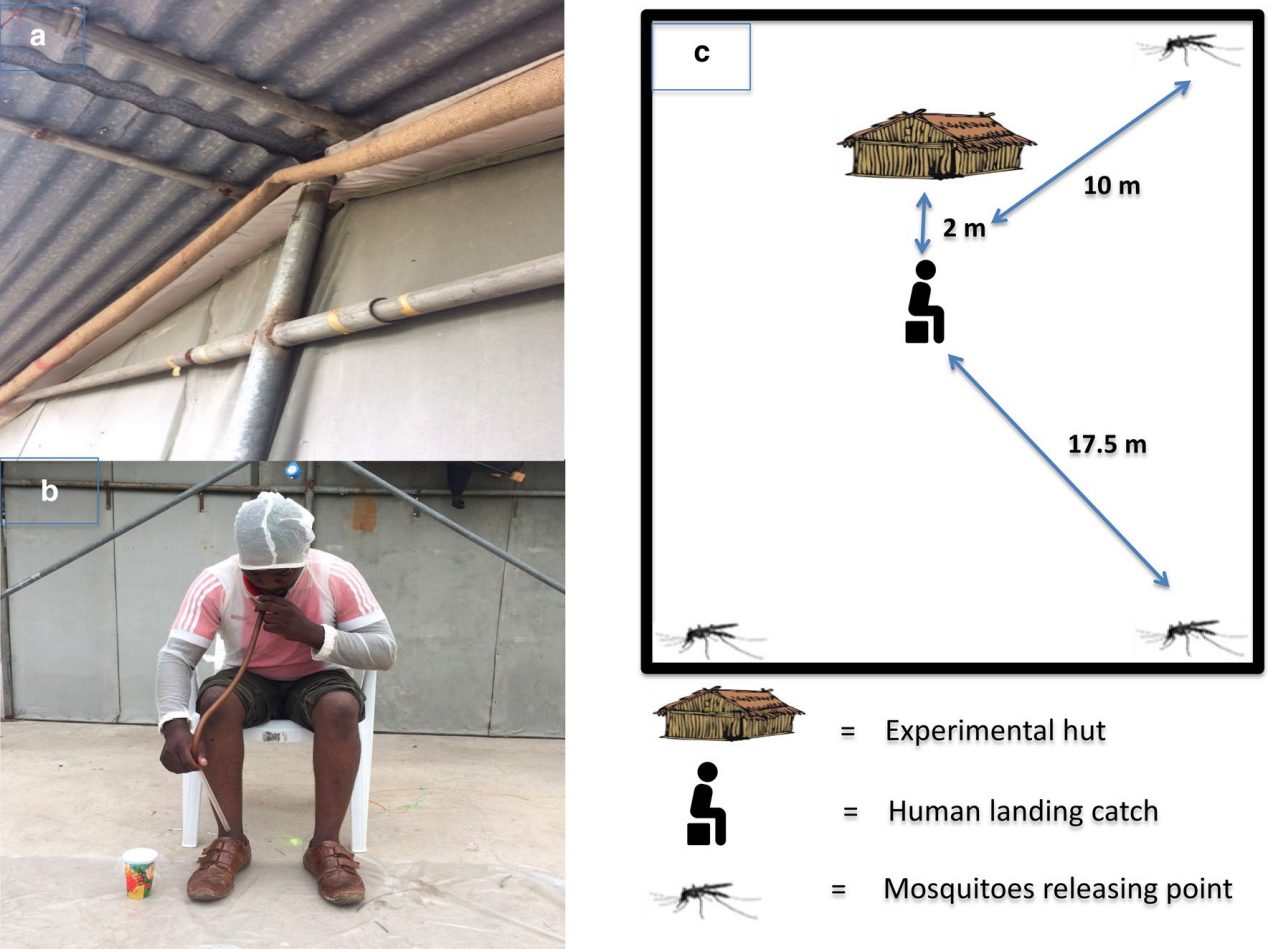
The evaluation of transfluthrin EPTI in the semi-field system. **a** Yellowish strips represent transfluthrin hessian strip position on the eave “EPTI”. **b** A volunteer sitting outside the experiment hut conducting HLC. **c** The schematic representation of the experiment inside a compartment of the semi-field system

The primary aim of the study was to determine if pyrethroid resistance in mosquitoes has a negative impact on the efficacy of transfluthrin EPTI. To do this, the treated hessian was placed on the eaves gaps of experimental huts located in the SFS, out of direct sunlight (Fig. 1a). Applying insecticide in this targeted way exploits the natural movement of air rising inside houses and being funnelled out through the eaves, over the treated hessian and into the peridomestic space, helping to disperse insecticide.

Human landing catch (HLC) were conducted 2 m outside the experimental hut (Fig. 1b, c) to mimic the peridomestic environment. Mosquitoes were released outside the experimental hut at every corner of the SFS compartment, eliminating directional bias in their approach to the human volunteer. Three separate experiments were conducted to evaluate the efficacy of (1) TG transfluthrin EPTI against Ifakara strain, Mbita strain and Kingani strain mosquitoes; (2) EC transfluthrin EPTI against Ifakara strain, Mbita strain and Kingani strain mosquitoes; and (3) EC transfluthrin EPTI against Kisumu strain and Kisumu-kdr strain mosquitoes.

During each experiment, either transfluthrin EPTI or the control (water-treated hessian) was assigned to one of two separate compartments of the SFS. The treatments remained fixed for a block of four days, after which they were rotated. HLC volunteers rotated between compartments daily. Four volunteers were recruited but only two used each day. The experiment was conducted for 4 blocks over 16 days, after which each volunteer conducted HLC for each treatment 4 times in each compartment. The volunteers were rotated to control for any bias caused by individual attractiveness to mosquitoes [25]. Prior to the start of the experiment, for acclimatization, mosquitoes were transferred from the insectary to the middle compartment of the SFS 30–45 min before their release.

Each day 80 mosquitoes of each strain were introduced into each compartment. Mosquitoes were separated into batches of 20 per strain and placed into 4 release cages, one in each corner of each compartment. The mosquitoes were released remotely by gently pulling strings connecting the release cages to simulate mosquitoes approaching the peridomestic space from multiple directions.

Throughout the experiment, volunteers wore shorts, covered shoes, and bug jackets to standardize the area available for mosquito landings. Mosquitoes that landed on the area between the ankle and the knee were collected using mouth aspirators through HLC (Fig. 1b). Mosquitoes were recaptured continuously for 50 min every hour for 4 consecutive hours between 18:30 and 22:30 h. Each hour, a new collection cup was used and labelled with the time and date. These mosquitoes were transferred to the insectary after 4 h, supplied with 10% sucrose and held for 24 h to observe 24-h mortality.

### Sample size

Sample-size calculations were performed using simulation-based power analysis [25] in R statistical software version 3.02 (http://www.r-project.org) with a significance level of 0.05 for rejecting the null hypothesis. Data analysis for experimental data was planned to be conducted using generalized linear mixed models (GLMMs) [38]. Therefore, 1000 simulations of GLMMs approximating those used to analyse project data were run using a 2 × 2 Latin square design with volunteers rotating nightly. The power to predict the difference in mosquito landings between control and treatment was estimated as the proportion of the 1000 simulated data sets in which the null hypothesis was rejected when the GLMM was run. The simulations indicated that with an estimated 80 mosquitoes released per compartment per night and 60% recapture of released mosquitoes, there was 100% chance of detecting a 50% reduction in mosquito landings in the treatment arm after 16 nights of experimentation. Inter-observational variance among daily experiments was set at 5%, and variability between times based on previous experiments was set at 25%.

### Data analysis

Data were recorded on paper forms and double entered into Microsoft Excel. Cleaning and analysis were done in Stata 13 (StataCorp). For the WHO insecticide susceptibility tests, data were summarized as mean percentage (%) 24-h mortality of the four replicates and reported with 95% confidence intervals.

Data for each experiment using each transfluthrin formulation (EC or TG) were analysed separately (Additional files 1, 2 and 3). For the analysis of the data on the effect of TG transfluthrin EPTI against Ifakara strain, Mbita strain and Kingani strain mosquitoes the additional file 1 was used whereas for the EC EPTI against Ifakara strain, Mbita strain and Kingani strain the additional file 2 was used and the additional file 3 was used for the analysis on the effect of EC EPTI against Kisumu susceptible and KDr strains.

The relative effect of transfluthrin on HLR and 24-h mortality for different mosquito strains was investigated using GLMM with binomial distribution. For HLR, the dependent variable was the proportion of released mosquitoes that were recaptured. For mortality, the dependent variable was the recaptured proportion that died. Treatment, mosquito strain, compartment and volunteer were included as fixed categorical variables, with day included as a random effect. An interaction term between mosquito strain and treatment was included to determine if the effect of treatment varied between mosquito strains.

The protective efficacies of the transfluthrin EPTI against each mosquito strain were calculated as

$${\text{Protective}}\;{\text{efficacy}}\left( {{\text{PE}}} \right) = \left[ {\left( {{\text{C}}{-}{\text{T}}} \right)/{\text{C}}} \right] \times 100\% ,$$

where *C* stands for the number of mosquitoes landing in the control and *T* for the number of mosquitoes landing in the treatment. The PE was calculated for each day, and the mean proportion of mosquitoes landing was reported with 95% confidence intervals (CI). For 24-h mortality, the control-corrected mortality was calculated using Abbott’s formula [39]:

$${\text{Control}}\;{\text{Corrected}}\;{\text{mortality}} = \left( {{\text{T}}{-}{\text{C}}} \right)/\left( {1{-}{\text{C}}} \right) \times 100\% ,$$

where *C* and *T* represents percentage mortality among mosquitoes landing in the control and treatment, respectively. The control-corrected mortality was calculated for each day, and the mean percentage dead was reported with 95% CI.

## Results

### WHO insecticide susceptibility tests

The susceptibility status of each mosquito strain to permethrin and deltamethrin is presented in Table 1. *Anopheles gambiae* Ifakara and Kisumu susceptible strains were fully susceptible. *Anopheles arabiensis* (Kingani) and *An. arabiensis* (Mbita) strains were resistant to pyrethroids while *An. gambiae kdr* was resistant to only permethrin.

**Table 1:** KD and 24-h mortality of the malaria vectors tested during the WHO insecticide susceptibility test

Mosquitoes	Insecticides	Concentration (%)	24-h mortality^a^ (%) (95% CI)
Kisumu susceptible	Permethrin	(0.75)	100 (100–100)
	Deltamethrin	(0.05)	100 (100–100)
Kisumu-kdr	Permethrin	(0.75)	98.9 (95.8–100)
	Deltamethrin	(0.05)	100 (100–100)
Ifakara strain	Permethrin	(0.75)	100 (100–100)
	Deltamethrin	(0.05)	100 (100–100)
Mbita strain	Permethrin	(0.75	72.6 (59.9–87.9)
	Deltamethrin	(0.05)	71.1 (53.1–95.2)
Kingani strain	Permethrin	(0.75)	19.7 (10.1–38.6)
	Deltamethrin	(0.05)	24.4 (13.5–44.8)

^a^24-h mortality is defined as the proportion of dead after 24 h out of the total number of mosquitoes exposed. Proportion mortality is reported with 95% confidence interval

### The efficacy of the transfluthrin EPTI against different mosquito strains

In experiment 1 with TG transfluthrin, a significant interaction between strain and treatment was observed. This indicated that the effect of the transfluthrin EPTI varied between strains under investigation (*P* < 0.001; Table 2). The use of TG transfluthrin EPTI significantly reduced the odds of landing of pyrethroid-susceptible *An. gambiae* (Ifakara strain; OR = 0.22 [0.18–0.26], *P* < 0.001) and had a similar impact on the landing of highly pyrethroid-resistant *An. arabiensis* (Kingani; OR = 0.23 [0.19–0.27], *P* < 0.001; Table 3). However, while the TG transfluthrin EPTI reduced the landing of pyrethroid-resistant *An. arabiensis* (Mbita), it did so to a lesser extent (OR = 0.33 [0.28–0.39], *P* < 0.001; Table 3). When assessing the efficacy of the EPTI using PE, the PE was similar for susceptible Ifakara 46.2% (95% CI 45.6–65.5), moderately resistant Mbita 46.4% (95% CI 37.9–54.9) and the highly resistant Kingani strain 54.9% (95% CI 41.6–64.1; Table 3). The binomial GLMM for TG transfluthrin indicated that both volunteers 3 and 4 and compartment significantly influenced HLR (in both cases, *P* < 0.05; Table 2).

**Table 2:** Generalised linear model output estimating the effect of EC/TG transfluthrin and mosquito strain on human landing rate in the semi-field system, Bagamoyo, Tanzania

Variables	Experiment 1, TG transfluthrin- (5.25 g)		Experiment 2, EC transfluthrin (5.25 g)		Experiment 3, EC transfluthrin (5.25 g)	
	OR*	*P*-value	OR^a^	*P*-value	OR^a^	*P*-value
Treatment						
Control	1		1		1	
Transfluthrin	0.22 (0.18–0.26)	< 0.001	0.10 (0.08–0.12)	< 0.001	0.14 (0.12–0.17)	< 0.001
Strain (in control)						
Ifakara strain (susceptible)	1		1			
Mbita strain (metabolic)	0.43 (0.32–0.57)	< 0.001	0.34 (0.26–0.46)	< 0.001	–	–
Kingani strain (metabolic)	0.60 (0.45–0.80)	< 0.001	0.44 (0.33–0.59)	< 0.001	–	–
Kisumu susceptible	–	–	–	–	1	
Kisumu kdr	–	–	–	–	1.0 (0.86–1.17)	0.05
Volunteers						
Volunteer 1	1		1		1	
Volunteer 2	0.88 (0.69–1.14)	0.36	1.07 (0.84–1.39)	0.60	1.20 (0.99–1.46)	0.06
Volunteer 3	0.76 (0.59–0.98)	0.04	0.96 (0.74–1.24)	0.77	1.19 (0.98–1.44)	0.07
Volunteer 4	0.83 (0.72–0.95)	0.001	0.90 (0.78–1.04)	0.17	1.14 (0.94–1.36)	0.18
Compartment						
Compart 1	1		1		1	
Compart 2	0.90 (0.81–0.99)	0.04	0.79 (0.71–0.87)	< 0.001	0.93 (0.81–1.07)	0.30
Treatment * strain						
Transfluthrin * Ifakara strain	1		1			
Transfluthrin * Mbita strain	1.50 (1.18–1.91)	< 0.001	1.67 (1.29–2.17)	< 0.001	–	–
Transfluthrin * Kingani strain	1.03 (0.82–1.32)	0.75	2.29 (1.78–2.94)	< 0.001	–	–
Transfluthrin * Kisumu susceptible	–	–	–	–	1	
Transfluthrin * Kisumu kdr	–	–	–	–	0.98 (0.74–1.30)	0.90

^a^Odds ratio (OR) was adjusted for temperatures, humidity and all other variables in the table

**Table 3:** The adjusted odds ratio of mosquito landings and protective efficacy offered by EC and TG transfluthrin in the semi-field system, Bagamoyo, Tanzania

Transfluthrin EPTI	Mosquitoes	Landing in the presence EPTI		Landing in the control (reference)		Protective efficacy (% [95% CI])
		*n* (% landing [95% CI])^a^	OR (95% CI)	*n* (%landing [95% CI])^a^	OR (95% CI)	
TG	Ifakara strain	500 (39.0 [32.9–45.2])	0.22 (0.18–0.26)*	939 (73.4 [66.9–79.8])	1	46.2 (45.6–65.5)
	Mbita arabiensis	370 (29.5 [24.4–34.7])	0.33 (0.28–0.39)*	706 (55.2 [51.7–58.6])	1	46.4 (37.9–54.9)
	Kingani strain	378 (28.9 [22.4–35.4])	0.23 (0.19–0.27)*	804 (62.8 [56.4–69.2])	1	54.9 (41.6–64.1)
EC	Ifakara strain	341 (26.6 [21.2–32.1])	0.17 (0.14–0.20)*	980 (76.6 [70.3–82.9])	1	65.0 (57.0–72.2)
	Mbita arabiensis	224 (17.5 [12.2–22.8])	0.23 (0.19–0.27)*	697 (54.5 [51.9– 57.0])	1	67.6 (57.6–77.6)
	Kingani strain	347 (27.1 [20.5–33.7])	0.57 (0.42–-0.78)*	774 (60.5 [56.6–64.4])	1	55.6 (45.6–65.5)
EC	Kisumu susceptible	166 (12.9 [9.6–16.3])	0.14 (0.12–0.17)*	647 (50.5 [50.0–51.0])	1	74.3 (67.7–80.9)
	Kisumu kdr	164 (12.8 [9.6–16.0])	0.14 (0.11–0.17)*	648 (50.6 [50.1–51.1])	1	75.1 (69.2–82.2)

^a^Numbers in the control and treatment refer to the total number of mosquitoes caught/released during each experiment; the percentage recaptured is in bracket. The percentage landing was calculated by dividing the number recaptured (*n*) by the total released (*N* = 1280). The OR is adjusted for temperature, humidity, compartment, volunteers and all other factors in the table

*P-value < 0.05

In experiment 2, using EC transfluthrin EPTI, there was again a significant interaction between strain and treatment, although a different trend was observed (Table 2). As with TG, the EC transfluthrin EPTI was observed to reduce the odds of landing of susceptible *An. gambiae* (Ifakara strain; OR = 0.17 [0.14–0.20], *P* < 0.001) and pyrethroid-resistant *An. arabiensis* (Mbita; OR = 0.23 [0.19–0.27], *P* < 0.001). However, EC transfluthrin showed lower efficacy against *An. arabiensis* (Kingani; OR = 0.57 [0.42–0.78], *P* < 0.001; Table 3). The model also indicated that compartment significantly influenced HLR of the mosquitoes (OR = 0.79 [0.71–0.87], *P* < 0.001). None of the volunteers influenced HLR (*P* > 0.05; Table 2).

Finally, in the analysis of the data from experiment 3, the interaction was not significant with Kisumu susceptible and *kdr* strains, indicating that the transfluthrin EPTI reduced landings of the two mosquitoes species in the same way (Table 2). The odds of landing of Kisumu susceptible and Kisumu *kdr* were equally reduced (OR = 0.14 [0.11–0.17], *P* > 0.001; Table 3). During the experiments, the average temperature was 27.8 °C (23.8–31.5 °C) and average relative humidity (RH) was 76.5% (63.6–92%).

### Effect of species on HLR in the control

The effects of mosquito species on HLR were examined in the control. The two species colonized from wild mosquitoes in Ifakara, Tanzania, were compared. In both experiments, consistently higher catches were observed with the Ifakara strain than with the Kingani strain. For example, in experiment 2, *An. gambiae* s.s. (Ifakara) showed a higher landing proportion, with an average of 76.6% (95% CI 70.3–82.9) recapture, than did *An. arabiensis* (Kingani), with an average of 60.5% (95% CI 56.6–64.4) recapture, and this difference was significant (OR = 0.5 [95% CI 0.4–0.6], *P* < 0.001; Table 4).

**Table 4:** The adjusted odds ratio of mosquito landings and protective efficacy offered by EC and TG transfluthrin in the semi-field system, Bagamoyo, Tanzania

Transfluthrin EPTI	Mosquitoes	Landing in the presence of EPTI		Landing in the control	
		n (% landing [95% CI])^a^	OR (95% CI)	n (% landing [95% CI])^a^	OR (95% CI)
TG	Ifakara strain	500 (39.0 [32.9–45.2])	1	939, (73.4 [66.9–79.8])	1
	Mbita arabiensis	370 (29.5 [24.4–34.7])	0.65 (0.49–0.86)*	706 (55.2 [51.7–58.6])	0.43 (0.32–0.57)*
	Kingani strain	378 (28.9 [22.4–35.4])	0.62 (0.47–0.83)*	804 (62.8 [56.4–69.2])	0.60 (0.45–0.80)*
EC	Ifakara strain	341 (26.6 [21.2–32.1])	1	980 (76.6 [70.3–82.9)	1
	Mbita arabiensis	224 (17.5 [12.2–22.8])	0.58 (0.43–0.78)*	697 (54.5 [51.9–57.0])	0.34 (0.25–0.46)*
	Kingani strain	347 (27.1 [20.5–33.7])	1.01 (0.76–1.38)	774 (60.5 [56.6–64.4])	0.44 (0.33–0.59)*
EC	Kisumu susceptible	166 (12.9 [9.6–16.3])	1	647 (50.5 [50.0–51.0])	1
	Kisumu kdr	164 (12.8 [9.6–16.0])	0.99 (0.78–1.24)	648 (50.6 [50.1–51.1])	1.00 (0.86–1.17)

^a^Numbers in the control and treatment refer to the total number of mosquitoes caught/released during each experiment; the percentage recaptured is in bracket. The percentage landing was calculated by dividing the number recaptured (*n*) by the total released (*N* = 1280). The ORs are adjusted for temperature, humidity, compartment, volunteers and all other factors in the table

*P-value < 0.05

### Comparison of 24-h mortality induced by transfluthrin-treated eave ribbon between mosquito strains

At 5.25 g dosage, no significant difference in 24-h mortality was observed in the presence of transfluthrin EPTI compared to the control across all mosquitoes strains (*P* > 0.05).

## Discussion

### The efficacy of EPTI to reduce HLR of malaria vectors

This study was conducted to determine if pyrethroid resistance in mosquitoes would have a negative impact on the efficacy of transfluthrin EPTI. Findings showed that *An. arabiensis* Kingani strain mosquitoes expressing high phenotypical resistance to pyrethroids were less repelled than the moderately resistant Mbita strain when using EC transfluthrin. However, Kingani, Mbita and Ifakara strains were equally repelled when using TG transfluthrin. It is, therefore, unclear how the different levels of metabolic resistance affect the efficacy of transfluthrin EPTI. TG was less effective against Mbita than against the susceptible Ifakara strain (*An. gambiae*), while EC was less effective against both the Mbita and the Kingani strains (*An. arabiensis*). This may indicate that metabolic resistance is indeed detrimental to the efficacy of transfluthrin; however, it is important to be cautioned when comparing species that have different levels of human biting preference (*An. gambiae*, *An. arabiensis*) because it is unknown how this variation affects the efficacy of transfluthrin. This study used *An. gambiae* s.s. as a reference strain because colonization of the susceptible *An. arabiensis* strain was not possible due to widespread resistance.

This results suggest that *kdr* target site mutations do not reduce the efficacy of transfluthrin. However, this finding must be interpreted with caution because the susceptibility test of the mosquitoes used revealed low levels of phenotypic resistance. What is clear from this study is that, compared to the control, transfluthrin EPTI can reduce landings of resistant mosquitoes. These findings corroborate previous experiments conducted under field settings in Kilombero Valley, Tanzania [10, 11, 40], in which transfluthrin applied to hessian in eaves (at concentrations higher than 5.25 g) significantly reduced HLR by over 80% and as well in the SFS, where the PE was over 68% [41]. Andres et al*.* observed that transfluthrin-treated polyester strips provide significant protection in the semi-field using one species of mosquito that was moderately resistant to pyrethroid [41]. Furthermore, transfluthrin-treated eave ribbon provided protection in Kilombero Valley, where malaria transmission is transmitted by *An. arabiensis* and *Anopheles funestus* [42], which were confirmed to be highly resistant to pyrethroid [31].

Methodologies used by these previous experiments were not designed to directly compare the differences in HLR between pyrethroid-susceptible and resistant mosquitoes. This study, however, provides a unique opportunity to compare the efficacy of transfluthrin applied as EPTI across different mosquito strains expressing different types and levels of insecticide resistance. Much more work is needed in this area, looking at a wider range of mosquito strains and resistance mechanisms.

It is known that the structural differences between VPs such as transfluthrin, which contain tetrafluorobenzyl alcohol, and non-VPs, such as permethrin, which contain phenoxybenzyl alcohol, may explain the efficacy of transfluthrin against resistant mosquitoes [43]. Horstmann et al*.* observed that the enzyme responsible for detoxification of non-VPs is unable to bind to the tetrafluorobenzyl moiety of VPs, leaving them active against resistant mosquitoes [27]. Further work is needed to determine the mechanism that causes mosquitoes to be repelled by transfluthrin in order to ascertain whether cross-resistance is possible. On the other hand, combining multiple active ingredients in targeted eave applications may help to combat resistant mosquitoes. Strategies could also combine an SR with a chemical that has high-contact toxicity and thus kills those mosquitoes that are not repelled and that are attempting to enter through the eaves. It was observed that mosquitoes attempting to enter houses spend 80% of their time within 30 cm of the eave [44]; thus, adding a second AI may enhance the control of resistant vectors. As has been observed in one study where the addition of the synergist piperonyl butoxide (PBO) can enhance knockdown by mosquito coils treated with a VP [45].

Despite a reduction of the HRL due to EPTI, inconsistent findings were observed when using PE for measuring efficacy compared to the OR estimates from the model. Such difference may be explained by the fact that OR from the GLMM contains additional explanatory variables that are not considered when using the basic formula for PE calculation. It is, therefore, suggested that for the evaluation of spatial repellent in the semi-field system, GLMM estimates should be presented rather than the calculated PE. The GLMM estimates are more robust as they account for other variables.

### The effect of transfluthrin formulation on HLR

While the EC and TG formulations were not compared directly, the EC did produce higher reductions in HLR. This could be explained by formulation differences that may have resulted in higher release rates and thus in different amounts of transfluthrin available in the air. It is known that differential concentrations of transfluthrin will induce different behaviours, including avoidance, irritancy, knockdown and mortality [46]. This dosage-dependent difference in mosquito behavioural response is also observed in other pyrethroid insecticides, including deltamethrin, cyphenothrin, d‐tetramethrin and tetramethrin [47]. The practical advantage of using EC was that it readily dissolves in water, making it more convenient to use, whereas TG transfluthrin required emulsification with detergent to mix with water. Further investigation into transfluthrin formulations is needed to fully assess their efficacy.

### The influence of species and strain on HLR

**Fig. 2:**
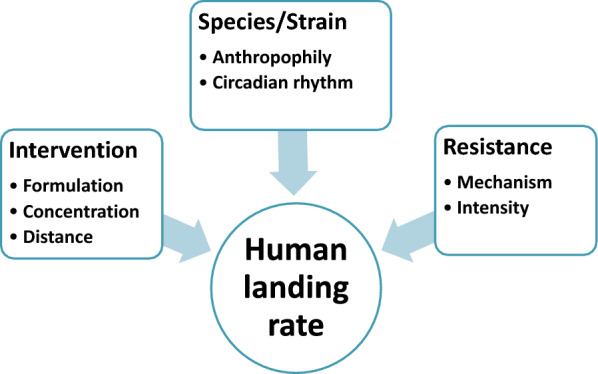
Factors shown to influence HLR and thus the protective efficacy of the EPTI

In addition to resistance, HLR was likely to be influenced by other factors (Fig. 2). In the absence of transfluthrin, this study observed differences in landing for the two different mosquito species. The Ifakara strain (*An. gambiae*) had a higher proportion of landing than did the Kingani strain (*An. arabiensis*) or the Mbita strain (*An. arabiensis*). Despite having been colonized for more than 10 years on particular Ifakara and Kingani strains, these mosquitoes demonstrated a behaviour seen in wild mosquitoes. Gilles et al*.* conducted an experiment in the field where they observed that *An. gambiae* s.s. were more likely than *An. arabiensis* strains to land on the person conducting HLC, indicating that species differences influence mosquito landing [48, 49]. The differences in landing between these mosquito species is caused by differences in attraction to human cues [48]. *Anopheles arabiensis* feed on both human and animals [50] depending on the relative abundance [51] or availability [52] of humans and animals, whereas *An. gambiae* s.s. feed exclusively on humans [53]. It is, therefore, suggested that the anthropophilic behaviour of *An. gambiae* s.s. may influence landing of these mosquitoes compared to the more opportunistic *An. arabiensis*.

Furthermore, the response of different species to VPs is well documented, with higher doses of transfluthrin needed to elicit escape responses in robust species such as *Aedes aegypti* than in *Anopheles* mosquitoes [46] and with different responses of members of the *Anopheles minimus* complex to pyrethroids and DDT [54]. It is also known that species vary in their sensitivity to topical repellents [55]. Therefore, in evaluating the efficacy of volatile pyrethroids, it is important to investigate the species and strains that will ultimately be targeted.

The difference in behavioural response of mosquitoes in the presence of repellent may also be associated with age. Studies have demonstrated that younger mosquitoes showed lower response to topical mosquito repellents [56], with very old mosquitoes being more responsive to repellents [57]. This study followed WHO guidance, using younger mosquitoes that are less likely to be affected by pyrethroid exposure [58]. Because the use of young mosquitoes may underestimate the PE of the VP, it is therefore recommended that further work be carried out on the optimal physiological age of mosquitoes to be used in studies of VP.

### 24-h mortality of malaria vectors after exposure to transfluthrin

The transfluthrin dose used in this study did not induce mortality for any of the mosquito strains; therefore, it was not possible to determine if there was cross-resistance between traditional pyrethroids and transfluthrin. Exposure to doses above 5.25 g of transfluthrin and long exposure have been associated with increased mortality in exposed mosquitoes [22, 59], so these higher doses would be required to determine if there is any difference between resistant and susceptible strains. Only those mosquitoes that were recaptured by HLC were examined for 24-h mortality; therefore, the full impact of transfluthrin on mortality cannot be measured. It is possible that those that did not land may have received a higher and potentially more lethal dose of transfluthrin. While it is useful to know if a mosquito will survive after a bite (and thus potentially go on to transmit disease), a better picture of the efficacy of VPs would be achieved if all mosquitoes were accounted for.

### Study limitation

This study has several limitations; firstly, currently, the CDC and WHO susceptibility bioassay do not have a recommended discriminating dose for testing transfluthrin in any mosquito species. Therefore, the resistance status of the mosquito colony was measured for traditional pyrethroids but not for transfluthrin. As transfluthrin has a different chemical structure there may be different mechanisms for resistance [27]. Therefore, future studies such as [60] are recommended to determine the discriminating dose of transfluthrin. Secondly, this study used only laboratory-reared mosquitoes, these mosquitoes may not represent the field mosquitoes and resistance mechanisms that may react differently to the transfluthrin spatial repellent. While field studies with transfluthrin eave-ribbons have shown that they can be effective in areas of insecticide resistance [40], further work is recommended in different settings with different resistance mechanisms and species. Thirdly, the experiment was conducted on susceptible and resistant mosquitoes from different species. It would have been advantageous to have susceptible and resistant mosquitoes of the same species to allow better approximation of the impact of resistance on resistant strains as the level of anthropophily of the different strains clearly influenced the results. Fourthly, in the semi field system, the wind was not detected. Under field conditions airflow (wind) might influence the efficacy of the push–pull system.

## Conclusion

Transfluthrin EPTI offered protection against all mosquito species regardless of the mosquitoes’ level of resistance. However, the differences in effect observed in different mosquitoes species highlight the fact that resistance in mosquitoes may be detrimental to the efficacy of transfluthrin. These findings demonstrated that transfluthrin-treated EPTI could be used to control malaria in areas with pyrethroid-resistant mosquitoes. This is particularly important in areas where transfluthrin will be considered for the control of mosquito vectors [20]. Although this study suggests that EPTI reduces HLR for both mosquitoes, additional evidence is needed to determine whether transfluthrin is effective against resistant mosquitoes and other species, such as *An. funestus*, where it is the dominant vector.

## Supplementary Information

**Additional file 1.** Dataset for the evaluation of TG EPTI against Ifakara strain, Mbita strain and Kingani strain that support the conclusion of this article.

**Additional file 2. **Dataset for the evaluation of EC EPTI against Ifakara strain, Mbita strain and Kingani strain that support the conclusion of this article. 

**Additional file 3.** Dataset for the evaluation of EC EPTI against Kisumu susceptible strain and Kisumu KDR strain that support the conclusion of this article.

## Data Availability

Data generated and analysed for this study are included in this article and its Additional files 1, 2 and 3.
